# Quantification of confocal fluorescence microscopy for the detection of cervical intraepithelial neoplasia

**DOI:** 10.1186/s12938-015-0093-6

**Published:** 2015-10-24

**Authors:** Fahime Sheikhzadeh, Rabab K. Ward, Anita Carraro, Zhao Yang Chen, Dirk van Niekerk, Dianne Miller, Tom Ehlen, Calum E. MacAulay, Michele Follen, Pierre M. Lane, Martial Guillaud

**Affiliations:** Imaging Unit, Department of Integrative Oncology, British Columbia Cancer Research Centre, Vancouver, BC V5Z 1L3 Canada; Department of Electrical Engineering, University of British Columbia, Vancouver, BC V6T 1Z4 Canada; Department of Pathology, British Columbia Cancer Agency, Vancouver, BC V5Z 4E6 Canada; Division of Gynaecologic Oncology, Department of Obstetrics and Gynaecology, University of British Columbia, Vancouver, BC V5Z 1M9 Canada; Brookdale University Hospital and Medical Center, Brooklyn, NY 11212 USA

**Keywords:** Confocal fluorescence microscopy, Cervical intraepithelial lesions, Image analysis, Colposcopy

## Abstract

**Background:**

Cervical cancer remains a major health problem, especially in developing countries. Colposcopic examination is used to detect high-grade lesions in patients with a history of abnormal pap smears. New technologies are needed to improve the sensitivity and specificity of this technique. We propose to test the potential of fluorescence confocal microscopy to identify high-grade lesions.

**Methods:**

We examined the quantification of ex vivo confocal fluorescence microscopy to differentiate among normal cervical tissue, low-grade Cervical Intraepithelial Neoplasia (CIN), and high-grade CIN. We sought to (1) quantify nuclear morphology and tissue architecture features by analyzing images of cervical biopsies; and (2) determine the accuracy of high-grade CIN detection via confocal microscopy relative to the accuracy of detection by colposcopic impression. Forty-six biopsies obtained from colposcopically normal and abnormal cervical sites were evaluated. Confocal images were acquired at different depths from the epithelial surface and histological images were analyzed using in-house software.

**Results:**

The features calculated from the confocal images compared well with those features obtained from the histological images and histopathological reviews of the specimens (obtained by a gynecologic pathologist). The correlations between two of these features (the nuclear-cytoplasmic ratio and the average of three nearest Delaunay-neighbors distance) and the grade of dysplasia were higher than that of colposcopic impression. The sensitivity of detecting high-grade dysplasia by analysing images collected at the surface of the epithelium, and at 15 and 30 μm below the epithelial surface were respectively 100, 100, and 92 %.

**Conclusions:**

Quantitative analysis of confocal fluorescence images showed its capacity for discriminating high-grade CIN lesions vs. low-grade CIN lesions and normal tissues, at different depth of imaging. This approach could be used to help clinicians identify high-grade CIN in clinical settings.

## Background

Cervical cancer represents a significant global cancer threat, particularly in low- and middle-income countries where the disease incidence is highest and cervical malignancies are the third leading cause of cancer death amongst women [1]. In 2007, there were more than 500,000 new cervical cancer cases worldwide and the number of cervical cancer deaths was 310,000 [2]. Improved testing accuracy and reduced screening costs could have significant positive impacts in developed nations with established cervical screening infrastructure. Although the Pap smear has reduced the incidence of cervical cancer worldwide [3], it’s low specificity results in an excessive number of colposcopy procedures performed, which in turn due to its low specificity, results in the acquisition of a large number of unnecessary biopsies.. The accuracy of colposcopy is highly dependent on the physician’s expertise. As shown in previous analyses, colposcopic examinations performs well in a diagnostic setting but poorly in a screening setting [4]. In particular, colposcopy has been reported to have a high sensitivity (96 %) and a low specificity (48 %) when differentiating abnormal tissues (squamous intraepithelial lesions (SILs)) from normal tissues (normal squamous epithelia and inflammation) [5].

There is room to improve the effectiveness of our current system that relies on histopathological review for colposcopically-guided biopsy specimens [6]. Optical measurements can be performed in a non-invasive manner to automatically identify CIN with high sensitivity and specificity, potentially reducing the frequency of unnecessary biopsies, and providing real time diagnosis with the possibility of immediate treatment by less experienced practitioners. In the last 10 years, new imaging and optical technologies have been developed to try to improve standard colposcopic examination of the uterus cervix, such as Optical Coherence Tomography [7] and Raman microscopy [8], Among these new technologies, confocal microcopy, either in reflectance or fluoresence mode, has been under development for almost two decades. In confocal reflectance microscopy, images represent the natural differences in refractive indices of cellular structures, whereas in confocal fluorescence microscopy, the tissue can be stained with a fluorescent contrast agent (fluorochrome) to improve the cellular contrast [9]. Precancerous lesions exhibits cell morphological and tissue architectural changes, including increased nuclear size, increased nuclear-cytoplasmic ratio, and decreased cell-to-cell distance [10]. Confocal microscopy is a non-invasive tool that can image epithelial tissues to provide information related to these epithelial changes. It does this by acquiring multiple images of cell nuclei at different focal depths. These images have sufficient contrast and resolution to allow the visualization of individual cells and nuclei, which; in turn, has driven the application of confocal microscopy to be useful in a variety of clinical contexts [9, 11–14]. Confocal microscopy has previously been used to study changes related to the grade of Cervical Intraepithelial Neoplasia (CIN) lesions. This work was pioneered by Dr Rebecca Kortum and her team at Rice University [15–18]. In these studies, features such as cell density, nuclear morphology (nuclear size, nuclear-cytoplasmic ratio), and tissue architecture (average distance between cells, etc.) were calculated from sample images to delineate disease states. With histopathology as the gold standard, these studies demonstrated the ability to discriminate high-grade dysplasia (CIN2 and CIN3) from low-grade dysplasia (HPV-associated changes and CIN1) at high sensitivity (86–100 %) and specificity (62–100 %). Later, another team confirmed these results using confocal endomicroscopy [19]. Four years ago, Kortum’s group has validated their technology and shown the same performance in vivo in clinical settings [20]. In the recent years, two other teams [21, 22] have also investigated and studied the feasibility of using endomicroscopy in clinical settings for screening or diagnostic purposes. Values of sensitivity and specificity of these studies to detect high-grade lesions, even in small cohorts of women, confirmed its potential for real time in vivo pathology [23]. Nevertheless, more work needs to be done to assess the true value of this technology in clinical settings. In the lats 10 years, our group has been facing numerous challenges, mostly related to instrumentation, implementation, quality control, and biological variability to implement reflectance and fluorescence spectroscopy in clinic [24]. We had underestimated the difficulty and complexity of moving optical technology from bench to clinic. We need to leverage this experience and better measure the capability of confocal microscopy. In this context, the objective of this work was to confirm and validate findings from previous studies with independent proprietary instrumentation as the ability of confocal microcopy to identify high-grade lesions and second to investigate the effect of varying imaging depth in the performance of our apparatus.

## Methods

**Fig. 1:**
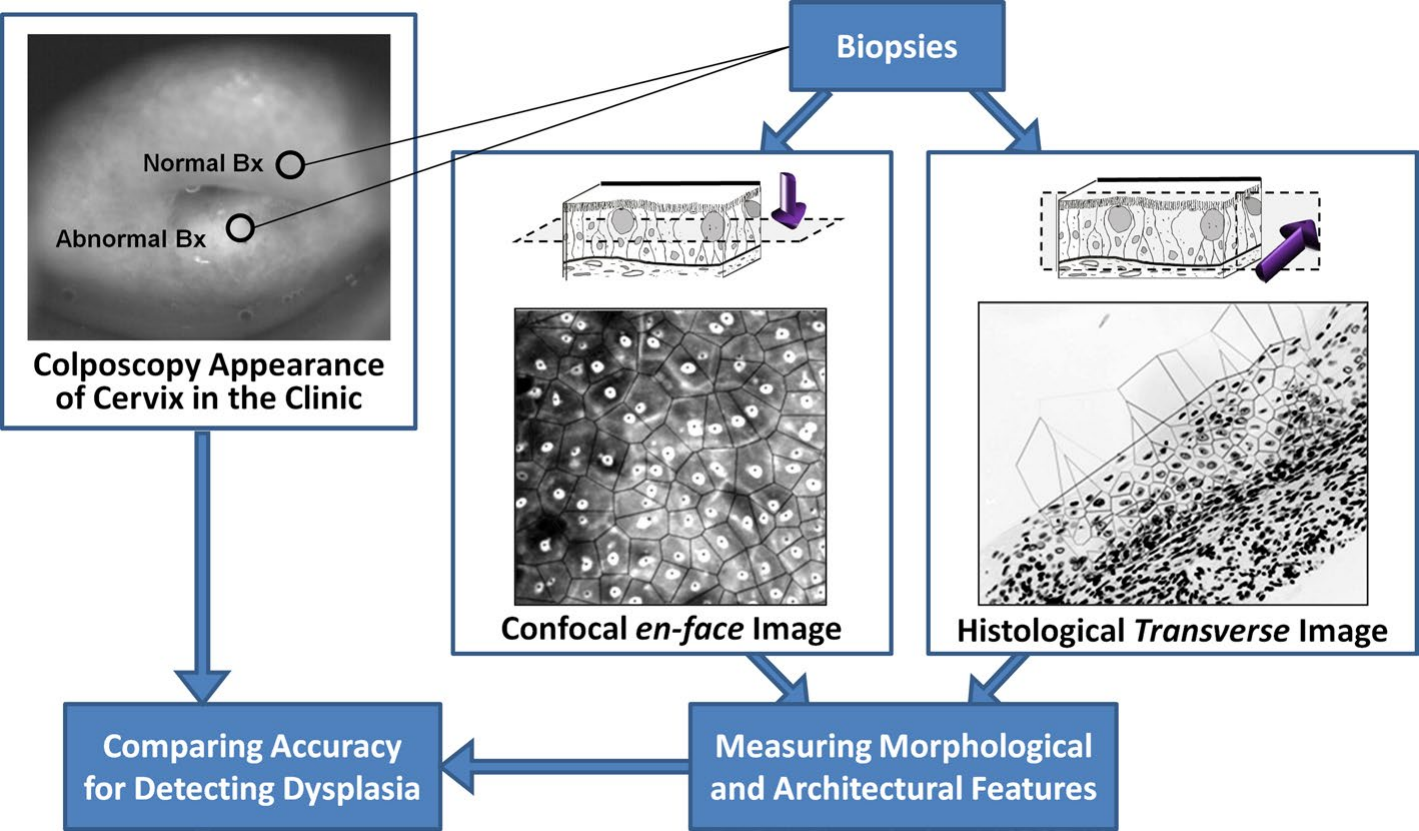
Study design. Based on the colposcopic impression, one biopsy is taken from an abnormal area and one biopsy from a normal area. Quantitative tissue phenotypic analysis of confocal fluorescence microscopy images and Feulgen-stained histological images is performed. Correlation between QTP features, colposcopic appearance and histopathological grades is investigated

Figure 1 outlines the design of the study described herein.

### Patient recruitment and specimen accrual

All patients gave informed consent, and the study was approved by the UBC BCCA Research Ethics Board and the Vancouver Coastal Health Research Institute (Protocols H09-03303 and H03-61235). We collected cervical biopsy specimens over a period of 22 months (April 2013 to February 2015) from baseline patients who were scheduled to have a colposcopic procedure performed at the Women’s Clinic at Vancouver General Hospital. Patients were referred to the clinic based on a prior abnormal Pap smear result. All cases were diagnosed as normal, low-grade CIN, or high-grade CIN [25]. Over a period of 62 clinic days, 143 patients were scheduled, and 50 patients were eligible and accepted to participate in this study (rate of recruitment = 34 %). Three of these patients were excluded from the study due to unsuccessful image acquisition. Forty-seven patients were successfully recruited, and normal and abnormal biopsies were collected from them. Participating patients were 20 to 51 years old with an average age of 31.7 years (SD = 8). None of these women were pregnant. In each case, following topical application of 5 % acetic acid to the cervix, normal and abnormal uterine cervix areas were identified by colposcopic examination by a trained gynecologist and biopsies were collected from these clinically selected sites. Biopsy specimens were immediately placed in saline and the colposcopic impression (normal, low-grade CIN, or high-grade CIN) was noted for each. Each biopsy was ~4 mm in diameter and ~2 mm in depth.

### Confocal fluorescence microscopy

Biopsies obtained in the clinic underwent confocal imaging at the British Columbia Cancer Research Centre (BCCRC) within 1 h of collection.. The biopsies were stained with Acriflavine fluorescence stain for 2 min while shaking on a shakerat low speed, as previously described [7]. The stain was prepared by dissolving 0.05 % Acriflavine Hydrochloride (Fluka) in 10 % phosphate-buffered saline. Biopsies were washed with saline for 1 min following staining. A 5 % solution of acetic acid was then added to each sample. The biopsies were placed on a microscope slide and cover-slipped. Confocal fluorescence microscopy was performed using a bench-top Carl Zeiss Axio Imager Z1 equipped with a custom laser-scanning confocal attachment. The custom confocal attachment employed a resonance scanner and galvanometer (Cambridge Technology) for laser scanning; an Avalanche photodiode (Hamamatsu) for detection; and a frame grabber (Matrox) to digitize the signal. Laser excitation was provided by a 488 nm laser (Coherent). All images were acquired using a 25X/0.80 water-immersion objective lens (lateral resolution of the system = 0.87 µm). The time frame from staining to completion of confocal imaging was approximately 10 min. Gray-scale confocal images were acquired and were 512 × 512 pixels in size. En face images were acquired every 5 µm. The first image of this image stack was acquired from just below the epithelial surface (z = 0 µm) while the depth of the last image (z = 80 µm) was limited by the working distance of the objective lens (250 µm) minus the thickness of the cover slip (170 µm).

### Confocal image analysis

**Fig. 2:**
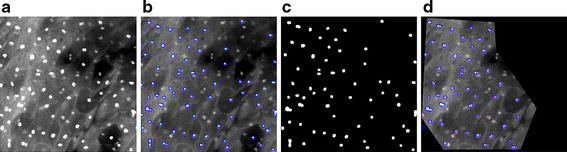
Image processing pipeline of confocal images using MATLAB algorithm. **a** Raw confocal image of a normal area; **b** edge detection by Canny algorithm; **c** nuclei segmentation; and **d** creation of nuclei masks followed by extraction of their centers of gravity

We developed an image processing algorithm in MATLAB (Release 2014b, The Mathworks Inc., Natick, MA, USA) to detect cell nuclei in confocal images (Fig. 2). This algorithm consisted of two steps: first, the derivative of a Gaussian filter was applied to compute the intensity gradient of the image and a Canny edge detector [26] was employed to detect edges of nuclei by finding local maxima of gradient (see Fig. 2b); second, thresholds were defined in Canny edge detector for detecting strong and weak edges and then a binary image of the edges, which mostly represent the nuclei boundaries, was obtained. An algorithm based on morphological reconstruction (*imfill* function in MATLAB) [27], and two sets of image erosion and image dilation were used to fill the nuclei boundaries and segment the nuclei (see Fig. 2c). The sequences of erosion and dilation were applied to remove the edges that do not represent nuclei boundaries. Then a Region of Interest (ROI) was selected on the confocal image to exclude unwanted noise that occurred at the time of imaging (e.g. bright, dark, defocused, or saturated regions on the image) (Fig. 2d). By reviewing the segmented objects (nuclei) in the ROI marked on the confocal image, we removed any objects that did not fulfill minimum quality requirements such as out of focus objects or objects with incorrect binary masks.

### Histopathological review

After confocal imaging, biopsies were fixed in formalin and transferred to BC Cancer Centre Pathology labfor sectioning.. Each biopsy was embedded in paraffin and nine 4 μm transverse sections were cut. Slides 1, 5, and 9 were stained with Hematoxylin and Eosin (H&E); and slide 2 was stained with Thionin–Feulgen stain, a stoichiometric stain for DNA [28]. The H&E stained sections were reviewed by an expert pathologist to establish disease grade. Once complete, all slides and results (including normal, reactive atypia, CIN1, CIN2, and CIN3) were returned to the study.

### Imaging of Thionin–Feulgen stained sections

The Thionin–Feulgen stained slides were scanned in absorbance mode using our *in*-*house*, high-resolution imaging system, Getafics [29]. This system consists of a 12-bit, double-correlated sampling MicroImager 1400 digital camera (pixels 6.8 μm^2^) placed in the primary image plane of the microscope with a 20X 0.75 NA Plan Apo objective lens (system resolution = 0.58 μm). For each Feulgen-stained slide, the worst histological diagnosis was found and imaged. Basal and superficial membranes were manually delineated, defining the ROI as described in another companion paper [30].

A semi-automatic, thresholding segmentation algorithm was used to detect cell nuclei located within the ROI. This thresholding algorithm separated objects (nuclei) from the background based on pixel intensity. Then, auto-focusing and an edge-relocation algorithm [31] was applied to the nuclei to precisely and automatically place the edge of the object at the region of highest local gray-level gradient. Digital gray-level images of the nuclei were stored in a gallery. All objects were manually reviewed by a technician to remove objects that did not fulfill the minimum quality requirements related to masking, focus, etc.

### Quantitative tissue phenotype (QTP) analysis

QTP analysis of histological and confocal images refers to the measurement of both the phenotype of the nuclei and the overall tissue architecture, as described below.

**Fig. 3:**
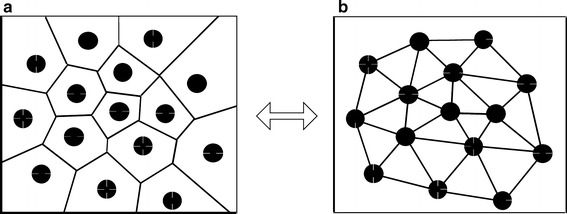
Voronoi tessellation of a set of points (**a**) and its dual Delaunay graph (**b**). Delaunay neighbors are connected if they share a common Voronoi edge

For all digitized nuclear images and recorded nuclear centers of gravity, we evaluated ~200 features associated with tissue architecture, nuclear and cellular shape, size, DNA amount, and chromatin texture organization [30, 32]. Based on preliminary work (unpublished data), we have restricted our analysis to four features (Table 1): (1) nuclear area; (2) cell density; (3) estimated nuclear-to-cytoplasmic (ENC) ratio; and (4) average distance between a nucleus and its three nearest Delaunay neighbors (3NDND). “Nuclear area” refers to the mean area of all segmented nuclei in µm^2^. “Cell density” refers to the number of nuclei per µm^2^. To calculate ENC ratio and 3NDND, we applied a Voronoi tessellation and Delaunay graphs, as follows. Given a set of points S (center of gravity of nuclei) in a plane, a Voronoi tessellation of the set S is the partition of the plane into polygons such that each polygon V(p) is associated with each point p of S. This is done in such a way that all locations inside V(p) are closer to p than to any other point in S (Fig. 3). The Voronoi polygon associated with a specific cell nucleus can be interpreted as an approximation of the cytoplasm of the cell [33]. The nuclear cytoplasmic ratio can then be approximated by the estimated nuclear-to-cytoplasmic (ENC) ratio, which is the ratio between the nuclear area and the Voronoi polygon area.

**Table 1:** Features generated from quantitative tissue phenotype analysis

Category	Feature name	Description
Nuclear morphology	Nuclear area	Mean area of the nuclei
Cellular morphology	ENC ratio	Nucleus area divided by the area of its associated Voronoi polygon.
Tissue Architecture	Cell density	Number of nuclei per mm^2^
	3NDND	Average distance between a nucleus and its 3 nearest Delaunay neighbors

The Delaunay diagram is the dual graph of the Voronoi diagram [34]. Two points of S are Delaunay neighbors if their Voronoi polygons share a common edge. A line segment joins each pair of Delaunay neighbors; the sum of these segments forms the Delaunay graph (see Fig. 3). Several features measuring the characteristics of the spatial distribution of the nuclei can be computed from the Delaunay graph. Based on previous studies (data not shown), only one of these features was selected for this analysis; this feature (3NDND) measures the average distance between a nucleus and its three nearest Delaunay neighbors (in µm).

All statistical significance was assessed using ANOVA and Fisher’s least significant difference (LSD) post hoc test was performed using STATISTICA software (StatSoft Inc., Tulsa, OK, USA).

## Results

**Fig. 4:**
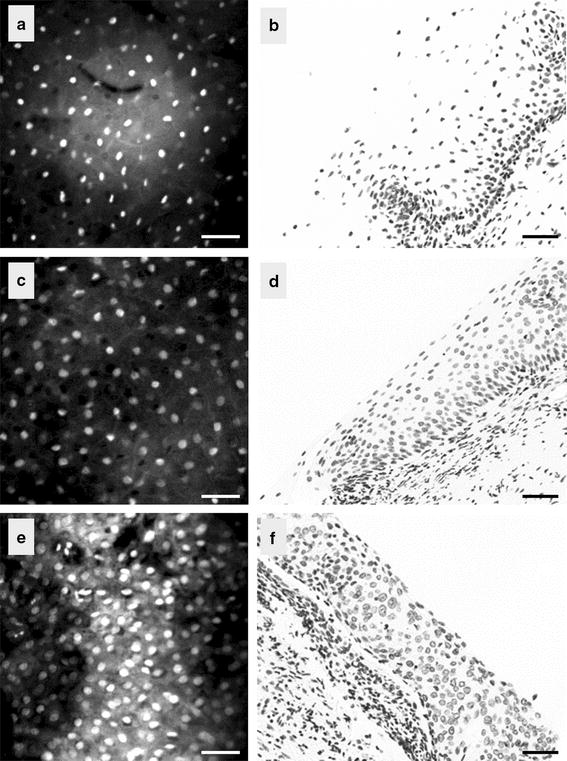
Confocal images of the cervix taken at a depth of 15 µm (*left*) and the corresponding histopathological section (*right*); **a**, **b** normal, **c**, **d** CIN1 lesion, and **e**, **f** CIN3 lesion. *Scale bar* is 50 µm

**Fig. 5:**
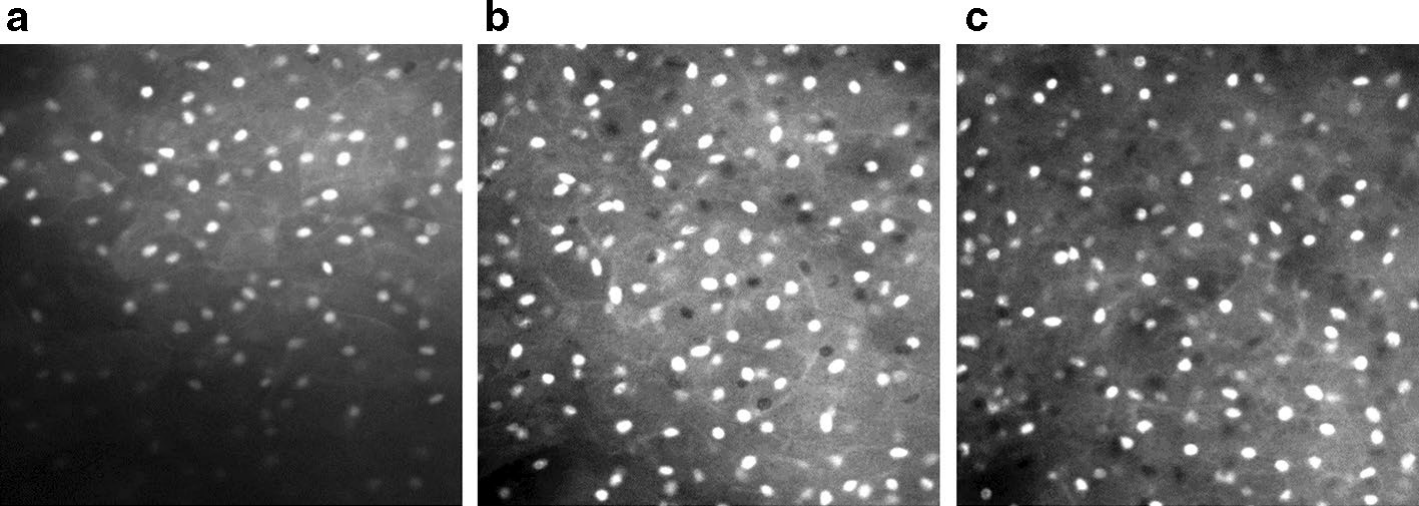
Confocal images of a normal area taken at **a** the epithelial surface; **b** at 15 µm below the epithelial surface; and at **c** 30 µm below the epithelial surface

**Fig. 6:**
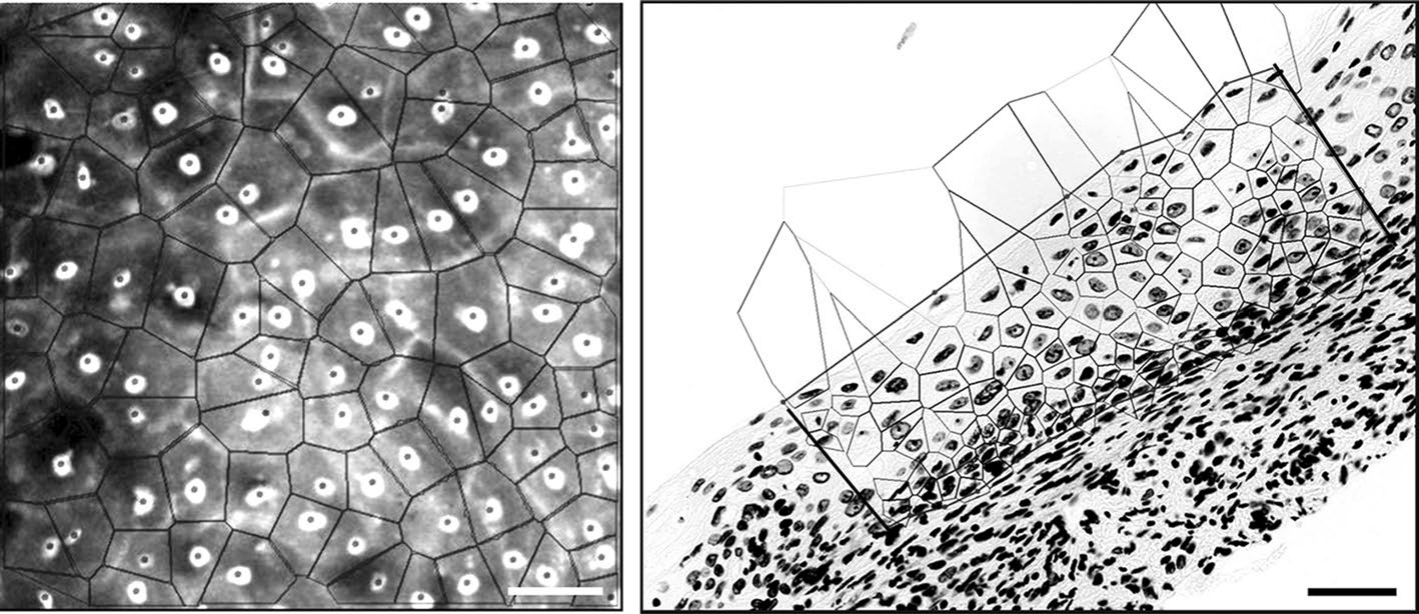
Voronoi tessellation overlaying the confocal image of a CIN1 lesion (*left*) and its corresponding histopathological image (*right*). *Scale bar* is 50 µm

Analyses were restricted to cases in which confocal images were of sufficient quality and histopathological review was completed. We considered three tissue groups: normal, low-grade CIN (CIN1), and high-grade CIN (CIN2 and CIN3). A total of 46 biopsies collected from 33 patients were used; 13 were classified as negative (i.e. normal), 18 as low-grade lesions, and 15 as high-grade lesions (9 CIN2 and 6 CIN3). Table 2 shows the confusion matrix between the colposcopic impression and the histopathological diagnosis. The results from two main analyses are presented in this section. First, we analyzed the confocal images imaged at a depth of 15 µm below the epithelial surface for all biopsies (as nuclei were clearly visible at this depth for all cases). Figure 4 shows the confocal and histological images of three cases. Second, to investigate the effect of depth of confocal imaging, we compared images of 21 specimens obtained at the surface of the epithelium, at 15 and at 30 µm below the epithelial surface. Figure 5 shows the confocal images of one specimen obtained at these three depths. Figure 6 shows the confocal and histological images with corresponding Voronoi tessellations for a CIN1 lesion.

**Table 2:** Classification of cervical biopsies based on colposcopic impression for the different histopathological grades

	Histopathology diagnosis				Total
	Normal	CIN1	CIN2	CIN3	
Colposcopic impression					
Normal	9	6	2	0	17
Low grade lesions	1	2	2	0	5
High grade lesions	3	10	5	6	24
Total	13	18	9	6	46

### Quantitative tissue phenotype analysis of confocal images

We studied the correlation between histology and QTP features of confocal images calculated from 46 cases at 15 µm below the epithelial surface. We observed that cell density was higher in high-grade CIN than in CIN1 or normal specimens (Fig. 4). We also observed that the penetration depth (maximum depth at which images can be obtained) was larger in normal biopsies than in abnormal biopsies (with an average of ~80 µm in normal cases vs. ~50 µm for abnormal cases—data not shown).

**Fig. 7:**
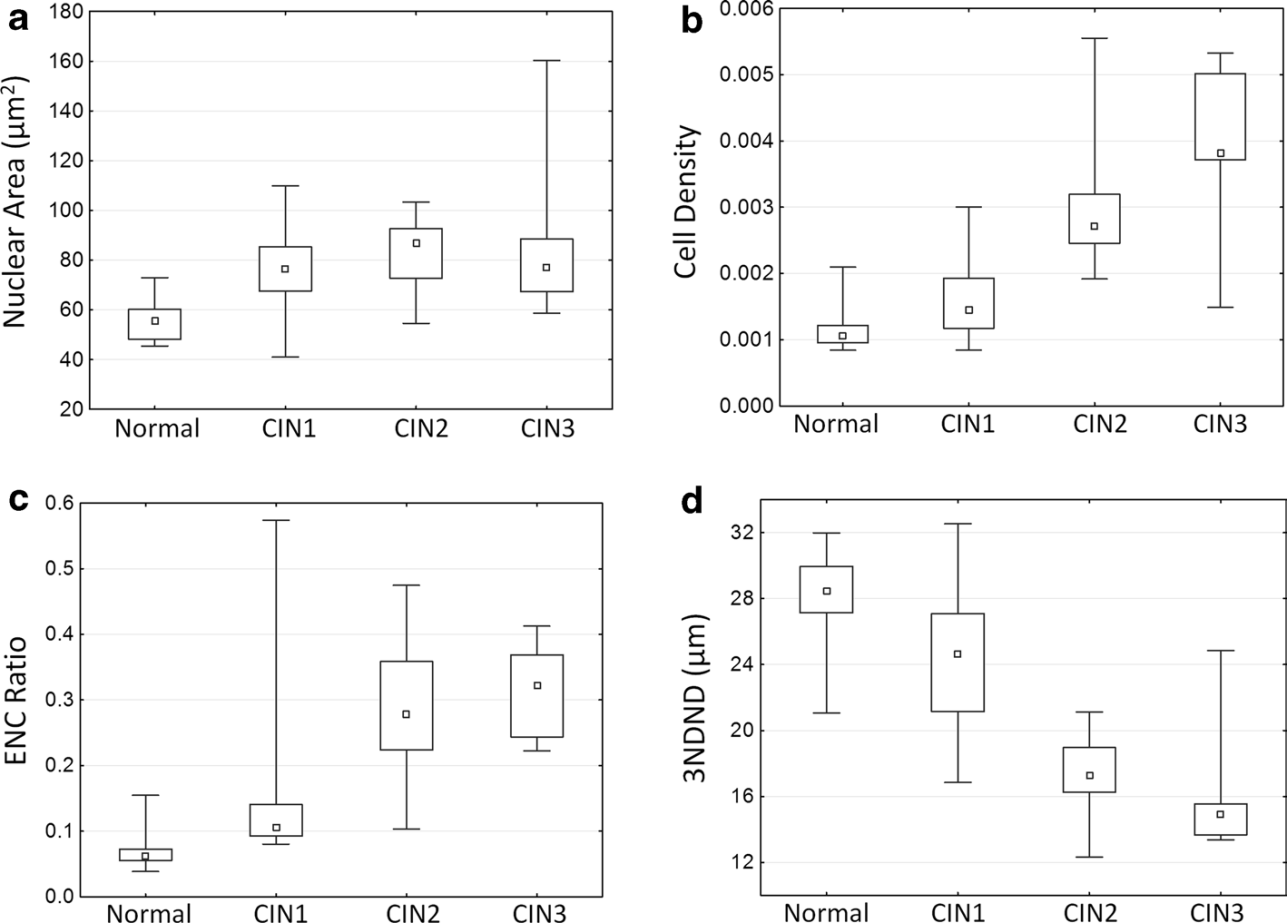
Analysis of confocal microscopy images. *Box-plot* distribution of QTP features for different histopathological diagnosis: **a** nuclear area; **b** cell density; **c** ENC ratio; and **d** 3NDND. (*central point* median; *box* first and third quartiles; *whiskers* 5 and 95 % quantiles)

**Fig. 8:**
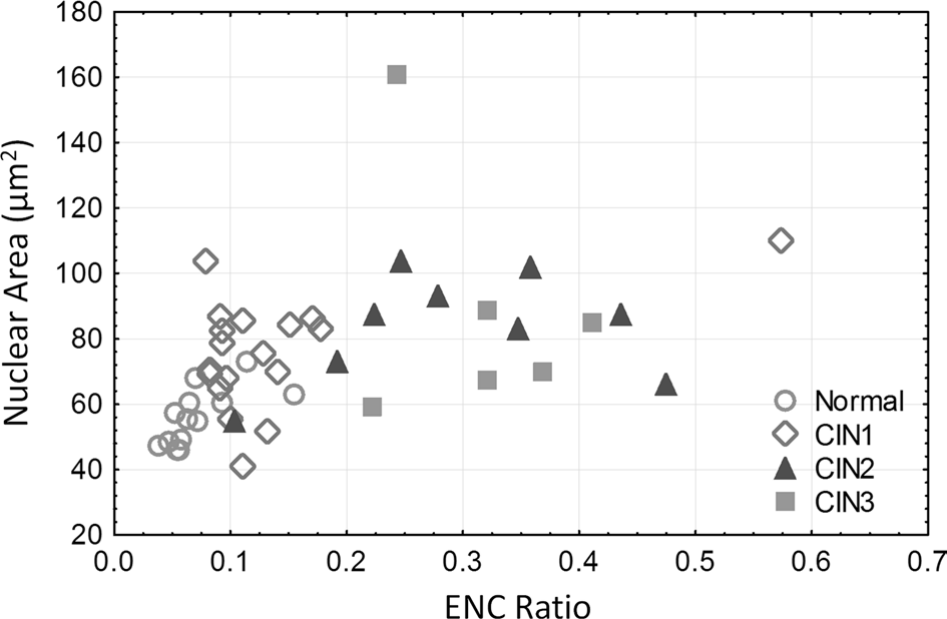
Analysis of confocal microscopy images. Relationship between ENC ratio and nuclear area for different histopathological grades

Figure 7 shows the distribution of nuclear area, cell density, ENC ratio, and 3NDND values for the three histological groups. The nuclei of normal specimens were significantly smaller than the nuclei of CIN1, CIN2, and CIN3 lesions (respectively p = 0.006, p = 0.002, and p = 0018) (Fig. 7a); in contrast, there were no differences observed between the different grades of dysplasia. Cell density increased regularly from normal to high-grade lesions with the cell density of high-grade lesions being twice as large as the cell density of low-grade lesions (Fig. 7b). Similarly, the ENC ratio increased from normal to high-grade lesions, reflecting an increase in the nuclear area relative to the cytoplasmic area (Fig. 7c). The distance between nuclei, as measured by 3NDND, decreased from normal to high-grade specimens (Fig. 7d). For these three features, the difference between low-grade and high-grade lesions were statistically significant (p = 2 × 10^−7^, p = 8 × 10^−6^, and p = 1 × 10^−6^, respectively). Neither cell density nor ENC ratio showed a statistical difference between normal and low-grade lesions (p = 0.136 and p = 0.052, respectively). However, there was a statistically significant difference in 3NDND between normal and low-grade lesions (p = 0.005). Figure 8 shows the scatter plot of the ENC ratio vs. the nuclear area for the different histopathological groups. There was a better separation seen between high-grade and low-grade lesions than between low-grade lesions and normal specimens.

### Quantitative tissue phenotype analysis of histological images

**Fig. 9:**
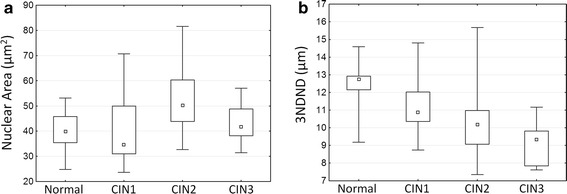
QTP analysis of histological images. *Box-plot* distribution of QTP features for different histopathological diagnosis: **a** nuclear area; and **b** 3NDND

Out of 46 biopsies we were able to successfully image 40 (12 normal, 15 low-grade CIN, and 13 high-grade CIN lesions). The other specimens were discarded from the analysis due to insufficient quality. Figure 9 shows the distribution of mean nuclear area and 3NDND for the different histopathological groups. There was no statistically significant difference in the nuclear area between the three groups (p = 0.12). On the other hand, 3NDND decreased regularly as dysplasia grade worsened. The difference was statistically significant between low-grade and high-grade lesions (p = 0.02), but not between normal and low-grade lesions (p = 0.08).

### Comparison of QTP analysis of confocal images with colposcopic appearance

**Fig. 10:**
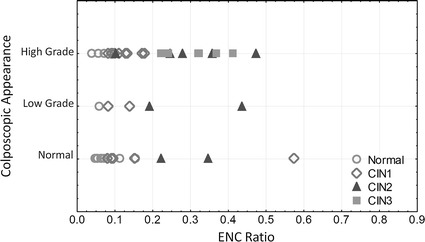
Analysis of confocal microscopy images. Relationship between ENC ratio and colposcopic appearance for different histopathological grades: (*circles* normal; *diamonds* CIN1; *triangles* CIN2; *squares* CIN3) (*central point* median; *box* first and third quartiles; *whiskers* 5 and 95 % quantiles)

We compared the QTP features with colposcopic impression, as identified by the clinicians. Figure 10 plots the relationship between colposcopic impression and ENC ratio calculated from confocal images (obtained at 15 µm) for different histopathological groups. We observe that 13 of the 31 colposcopically-defined high-grade CIN (41 %) were either low-grade CIN or normal cases, and 4 colposcopically-classified low-grade CIN were in fact high-grade lesions.

The Spearman correlation coefficient between histopathological diagnosis and colposcopic impression was 0.43. The correlation between histopatholgical diagnosis and nuclear area, cell density, ENC ratio, and 3NDND were 0.56, 0.77, 0.81, and 0.77, respectively.

To assess the potential of confocal fluorescence microscopy for detecting cervical dysplasia, we calculated the sensitivity and specificity of the ENC ratio and 3NDND values for detecting either high-grade lesions (i.e. CIN2 or CIN3) or all lesions (i.e. CIN1, CIN2, or CIN3) (Table 3). Based on the feature distribution illustrated in Fig. 7, biopsies were classified into three groups: normal if the ENC ratio was lower than 0.08, low-grade if the ENC ratio was larger than 0.08 and smaller than 0.18, and high-grade if the ENC ratio was higher than 0.18. Similarly, biopsies with a 3NDND larger than 25 µm were classified as normal; biopsies with a 3NDND value between 21 and 25 µm were classified as low-grade; and biopsies with a 3DDND value smaller than 21 µm were classified as high-grade. The sensitivity and specificity of colposcopic impression for detecting any grade of CIN was respectively 75 and 69 % (Table 3). The sensitivity and specificity of detecting any grade of CIN for both ENC ratio and 3NDND values were comparable to the values seen with colposcopic impression. The sensitivity and specificity of ENC ratio for detecting high-grade CIN alone were 93 and 96 %, respectively.

**Table 3:** Sensitivity and specificity of colposcopic appearance, ENC ratio, and 3NDND for the detection of cervical dysplasia (imaging depth is 15 µm)

	Detecting high-grade lesions		Detecting any lesion	
	Sensitivity (%)	Specificity (%)	Sensitivity (%)	Specificity (%)
Colposcopic impression	73	58	75	69
ENC ratio	93	96	100	84
3NDND	66	90	100	80

### Quantitative tissue phenotype analysis of confocal images at different depths

**Fig. 11:**
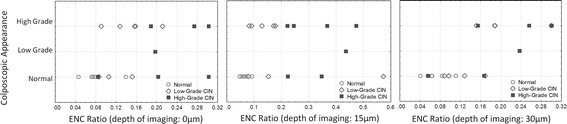
Analysis of confocal microscopy images. Relationship between ENC ratio and colposcopic impression at different imaging depths; at the epithelial surface (*left panel*), at 15 µm below the epithelial surface (*middle panel*); and at 30 µm below the epithelial surface (*right panel*)

To investigate the performance of our technology at different depths, we imaged 21 cervical biopsies at three different depths: at the epithelial surface, 15 µm beneath the surface, and 30 µm beneath the surface. Figure 11 shows the scatter plot of ENC ratios measured at these three depths vs. colposcopic impression. Table 4 shows the sensitivity and specificity of detecting dysplasia using either the ENC ratio or 3NDND measured on images, which were sampled at different depths from the epithelial surface. For the classification of biopsies based on their corresponding ENC ratio and 3NDND values, we applied the same criteria as described in “Comparison of QTP analysis of confocal images with colposcopic appearance” (e.g. biopsies with an ENC ratio <0.08, 0.08 < ENC ratio < 0.18, and ENC ratio >0.18 were respectively classified as normal, low-grade, and high-grade). The sensitivity and specificity of colposcopic impression for detecting high-grade CIN were respectively 57 and 64 %. The sensitivity and specificity of detecting any dysplasia using ENC ratio and 3NDND at any of the three depths were comparable to those of colposcopic appearance (Table 4). The sensitivity and specificity of the ENC ratio for detecting any dysplasia at 15 µm beneath epithelial surface was higher than the ones measured at the surface or at 30 µm.

**Table 4:** Sensitivity and specificity of ENC ratio and 3NDND for the detection of cervical dysplasia at different confocal imaging depths

Imaging depth	QTP features	Detecting high-grade lesions		Detecting any lesions	
		Sensitivity (%)	Specificity (%)	Sensitivity (%)	Specificity (%)
Surface	ENC ratio	85	92	100	83
	3NDND	71	85	66	83
15 µm	ENC ratio	100	92	100	83
	3NDND	100	85	80	83
30 µm	ENC ratio	50	78	92	66
	3NDND	83	71	78	66

Interestingly, we observed that low-grade lesions that showed a high ENC ratio value (measured at 15 µm [see Fig. 11b] and comparable to high-grade lesions) have an ENC ratio value similar to other low-grade lesions when imaged from the surface or 30 µm deep. In addition, we noticed that a confocal image obtained at a 15 µm depth was saturated due to the maladjustment of the parameters of confocal microscopy at the time of imaging, explaining the overestimation of the nuclear size.

## Discussion

Currently, colposcopic examination of the cervix is used to detect preneoplastic lesions after an abnormal Pap test and to guide biopsy selection for disease diagnosis and staging. Unfortunately, challenges exist with regards to the accuracy and reproducibility of this approach. Pre-neoplastic lesions are associated with a variety of morphologic and tissue architectural alterations; and confocal microscopy has previously been used to non-invasively detect changes in cell morphology and tissue architecture, which may be used to confirm histopathological diagnoses and assess progression likelihood [2, 11–13]. Ultimately, clinical application of confocal imaging will lead to a new breed of “clinical pathologists” who can detect and grade preneoplastic lesions in vivo using real-time optical imaging [35].

In this study, we collected confocal fluorescence images of fresh cervical biopsy specimens and calculated ~200 features (including morphologic and architectural) for 46 specimens using our automatic software (written in MATLAB). The results indicated that, two features, in particular, (ENC ratio and 3NDND values) could be used to classify dysplasia grade in confocal images. We demonstrated that these features were capable of differentiating high-grade CIN from normal and low-grade CIN with a high sensitivity and specificity as compared to results seem from colposcopic impression analyses (see Table 3 and previous studies [15, 17, 19]). We compared the scatter plot of nuclear area vs. ENC ratio presented in Fig. 8 with that of a previous study by Collier et al. [15], who studied the reflectance confocal microscopy images of normal and abnormal cervical biopsies obtained at a depth of 50 μm. These two scatter plots show a similar distribution of normal specimens, low-grade, and high-grade lesions. Our preliminary results suggest the potential for use of confocal fluorescence imaging as a tool in clinical settings for biopsy site selection. Currently, many unnecessary biopsy specimens are obtained in clinic due to the relatively low specificity of the colposcopy [5]. The use of confocal microscopy for guiding the biopsy excision process could lower the cost of unnecessary diagnostic procedures and, more importantly, improve the patients’ experience.

In addition, we have shown that quantitative features measured from confocal fluorescence images compare well with features calculated on histological images. These features are also consistent with classical descriptions of cervical dysplasia grading based on histopathological criterion of H&E-stained slides (e.g. increased nuclear-cytoplasmic ratio or decreased cell–cell distances [9]). Naturally, the apparent advantage of confocal images over histological images is that confocal images delineate cell structures in vivo, removing the need to acquire and process tissue specimens (which can be costly in terms of time and funds).

Images obtained in this study were of a higher resolution than those of previous studies done using reflectance microscopy [15, 17]. This may be due to the use of a contrast agent in confocal fluorescence microscopy, which improves the cellular contrast by staining the nuclei of the epithelium. In this study, Acriflavine dye was used to stain the nuclei for confocal fluorescence microscopy, a process that leads to clearly visible nuclei in resulting images [13]. However, the images obtained in this study were taken at a depth (from the epithelium surface) that was shallower than those of previous studies [15, 17]. Although Acriflavine strongly stained the superficial epithelial cell nuclei, the dye penetration into deeper layers was limited, as already observed by Tan et al. [19].

Fortunately, unlike in reflectance microscopy, nucleo-architectural features can be robustly measured even at such limited penetration depths. This is due to the fact that high-grade lesions are characterized by the presence of dysplastic cells in the superficial layers of the epithelium. Our results show that the optimal depth of imaging to discriminate high-grade lesions from other lesions is 15 µm. It was challenging, however, to differentiate normal tissue from mild dysplasia based on nuclear and cellular features at 15 µm below the surface of the tissue. In the future, additional experiments based on a larger sample size will be necessary to statistically establish the optical imaging depth for in vivo real time confocal microscopy use.

It should be noted that other studies that used deeper confocal imaging were also unable to accurately differentiate normal tissue from mild dysplasia [15, 18].

In conclusion, we showed that first, the ability to use only a few morphological and architectural features to differentiate normal tissue and low-grade lesions from high-grade dysplasia; and second, a similar performance was achieved at different epithelial depths (see Table 4). This is a significant finding since it is likely that assessing the exact depth of imaging in vivo will be challenging. Furthermore, by imaging and comparing features at different epithelial depths, we can reach a more accurate and reproducible quantification of dysplastic changes. Indeed, we can foresee that a clinical could use different depth information to increase his confident in case the images are not good enough, or as it happens in the cervix in the presence of keratin at the surface of the epithelium, or when the contact is not adequate. By moving up and down with the probe, our data has shown that results will be affected.

Our experimental design suffers from one limitation. In Feulgen-stained sections, an experienced cytotechnician carefully chooses the Region of Interest corresponding to the diagnostic area as defined by the study pathologist (Dr. van Niekerk) on the H&E section. This area corresponds to the worst dysplastic area present on the section. Toavoid any selection bias, the region of interest on confocal images were randomly selected without any input from the pathologist; this means it is possible that the confocal area does not exactly match the corresponding histopathological diagnosis and area selected by the pathologist. Unfortunately, this is a limitation of this study. Nevertheless, we believe that our approach is valid and justified as we are trying to study the average correlation between this technology and clinical appearance. By choosing random areas, we are more likely to fail to spot dysplastic areas when the lesion is small (low-grade) than when the lesion is large (high-grade), resulting in an underestimation of the “true” sensitivity of this technology for detecting low-grade lesions. In the future, we believe that a new breed of pathologists/clinicians will become skilled in selecting and identifying the precancerous lesions on confocal images. Our results may actually underestimate the power of confocal microscopy for detecting cervical dysplasia.

## Conclusion

We have studied the potential of nuclear-architectural features measured from ex vivo fluorescence confocal images of fresh cervical biopsy specimens. By correlating these features with histopathology diagnosis, we confirmed that confocal fluorescence microscopy can differentiate high-grade lesions from low-grade lesions and normal tissues. More importantly, we have shown that these results were comparable when images were collected at different depths. New experiments involving deeper tissue examinations by improved staining techniques will help to better differentiate normal tissues from low-grade lesions. This study serves as a critical step towards utilizing confocal approaches in an in vivo clinical application. This study was part of an ongoing project with the ultimate goal of integrating advances in cancer biology and optical technology to develop cost-effective tools to aid in early detection of cervical cancer [36].
